# Tumor volume: a new prognostic factor of oncological outcome of localized clear cell renal cell carcinoma

**DOI:** 10.1186/s12885-021-07795-8

**Published:** 2021-01-19

**Authors:** Shao-Hao Chen, Long-Yao Xu, Yu-Peng Wu, Zhi-Bin Ke, Peng Huang, Fei Lin, Xiao-Dong Li, Xue-Yi Xue, Yong Wei, Qing-Shui Zheng, Ning Xu

**Affiliations:** Department of Urology, the First Affiliated Hospital of Fujian Medical University, 20 Chazhong Road, Fuzhou, 350005 China

**Keywords:** Tumor volume, Oncological outcome, Prognostic factor, Survival, Localized clear cell renal cell carcinoma

## Abstract

**Background:**

Clear cell renal cell carcinoma (ccRCC) is one of the most frequent malignancies; however, the present prognostic factors was deficient. This study aims to explore whether there is a relationship between tumor volume (TV) and oncological outcomes for localized ccRCC.

**Methods:**

Seven hundred forty-nine localized ccRCC patients underwent surgery in our hospital. TV was outlined and calculated using a three-dimensional conformal radiotherapy planning system. We used receiver operating characteristic (ROC) curves to identified optimal cut-off value. Univariable and multivariable Cox regression models were performed to explore the association between TV and oncological outcomes. Kaplan-Meier method and log-rank test were used to estimate survival probabilities and determine the significance, respectively. Time-dependent ROC curve was utilized to assess the prognostic effect.

**Results:**

Log rank test showed that higher Fuhrman grade, advanced pT classification and higher TV were associated with shortened OS, cancer-specific survival (CSS), freedom from metastasis (FFM) and freedom from local recurrence (FFLR). multivariable analysis showed higher Fuhrman grade and higher TV were predictors of adverse OS and CSS. The AUC of TV for FFLR was 0.822. The AUC of TV (0.864) for FFM was higher than that of pT classification (0.818) and Fuhrman grade (0.803). For OS and CSS, the AUC of TV was higher than that of Fuhrman grade (0.832 vs. 0.799; 0.829 vs 0.790).

**Conclusions:**

High TV was an independent predictor of poor CSS, OS, FFLR and FFM of localized ccRCC. Compared with pT classification and Fuhrman grade, TV could be a new and better prognostic factor of oncological outcome of localized ccRCC, which might contribute to tailored follow-up or management strategies.

## Background

As one of the most frequent malignancies, renal cell carcinoma (RCC) accounted for approximately 2–3% of all cancers [1, 2]. There were 80–90% of clear cell RCC (ccRCC), which is the major histological subtype of RCC [3, 4]. Metastases occurred in about 25–30% of patients with ccRCC at first diagnosis; besides, 20–30% of localized RCC patients have metastases after treatment [5]. Hence, there is an urgent need to identify prognostic parameters predicting recurrence or metastasis.

Although Fuhrman nuclear grading [6] and TNM systems [7] are useful prognostic parameters, they are still not perfect [8]. Klatte et al. [8]. reported that Fuhrman nuclear grading system have been shown to be suboptimal due to its inter- observer and intra-observer variability. T staging is based on the maximum diameter of solid tumors such as RCC [9] and hepatocellular carcinoma [10]; however, tumor diameter could not be fully representative of tumor volume (TV). Other well-known prognostic parameters included tumor necrosis [11], warm ischemia time, multifocality [12], bilateral occurrence of carcinoma [13], sarcomatoid and rhabdoid features [14], vascular and lymphatic microfiltration [15], caval or renal thrombosis [16]; however, they were deficient. The insufficiency of present prognostic factors has resulted in consideration of new factors. TV is more representative of tumor burden compared with diameter. There is accumulating evidence that the measurement of TV is applied to the evaluation of renal function [17–20], but only a few report on the relationship between oncological outcomes and TV of ccRCC [21].

The objective of our study was to evaluate TV, clinicopathological features and oncological outcomes of localized ccRCC, to determine whether TV is a prognostic factor for cancer-specific survival (CSS), overall survival (OS), local recurrence and distant metastases of ccRCC.

## Methods

### Study population

**Fig. 1:**
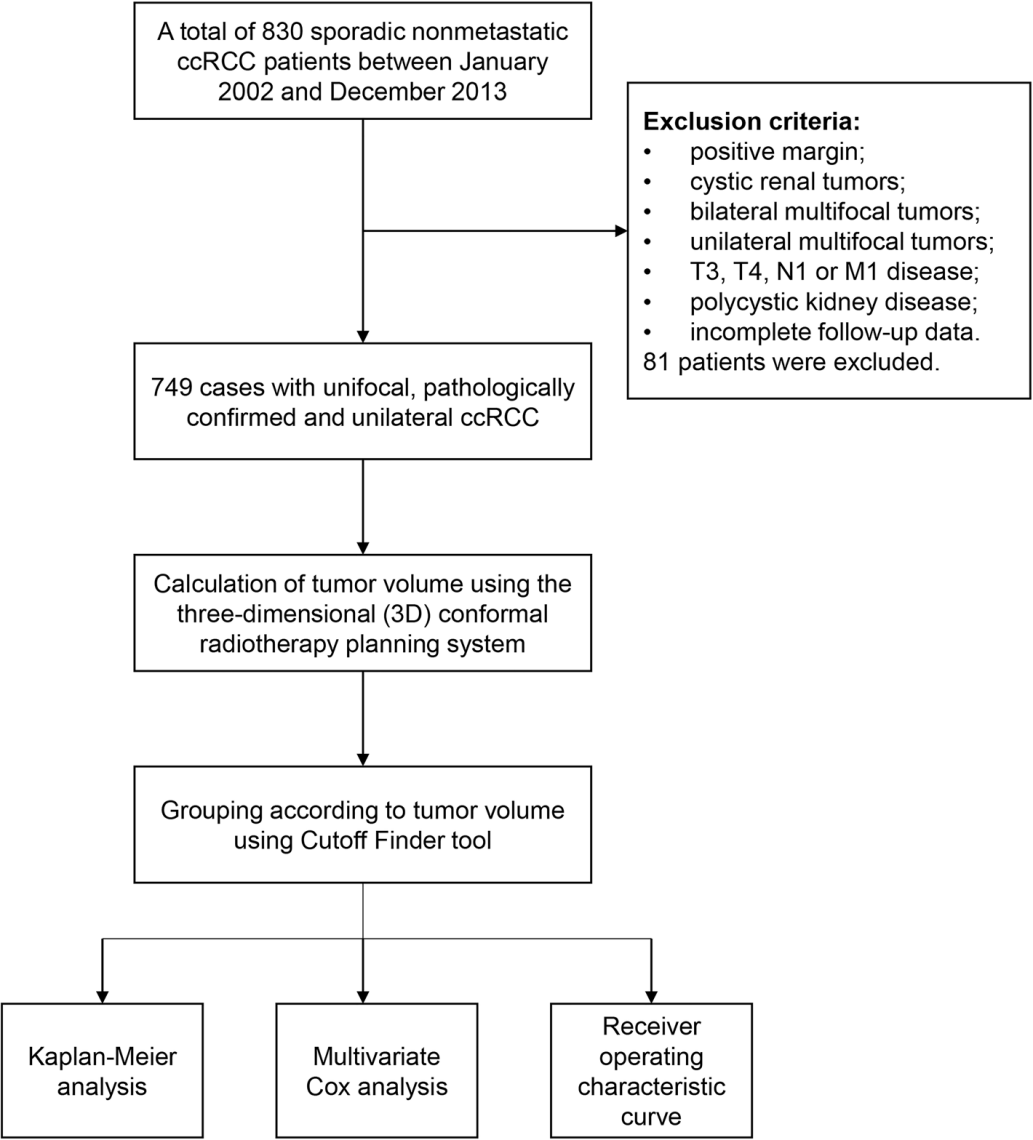
The flow chart of this study

The flow chart of this study is shown in Fig. 1. A total of 830 sporadic nonmetastatic ccRCC patients between January 2002 and December 2013 in our hospital were identified. All these patients underwent partial or radical nephrectomy. Only unifocal, pathologically confirmed and unilateral ccRCCs were included. We excluded patients with cystic renal tumors, bilateral multifocal tumors, unilateral multifocal tumors, patients with T3, T4, N1 or M1 disease, positive margin and also polycystic kidney disease. The symptomatic group included patients with abdominal masses, flank abdominal pain or hematuria while those who discovered only by computed tomography (CT), ultrasound, or magnetic resonance imaging were classified as the asymptomatic group. Clinicopathologic data such as age, gender, tumor diameter, tumor location, Fuhrman grade and TV were collected (Table 1).

**Table 1:** Patients and tumors characteristics

Variable	Mean ± SD or median (interquartile range) or n (%)
Age, year	54.72 ± 11.48
Tumor diameter, cm	5.94 ± 3.06
Gender	
Male	505 (67.4)
Female	244 (32.6)
Tumor location	
Left kidney	366 (48.9)
Right kidney	383 (51.1)
Hypertension	
No	488 (65.2)
Yes	261 (34.8)
Diabetes	
No	640 (85.4)
Yes	109 (14.6)
Smoking history	
No	430 (57.4)
Yes	319 (42.6)
BMI	
< 25	507 (67.7)
≥ 25	242 (32.3)
Sugical approach	
Open	321 (42.9)
Laparoscopic	428 (57.1)
Surgical modality	
Partial nephrectomy	317 (42.3)
Radical nephrectomy	432 (57.7)
Tumor necrosis extent	
TN = 0%	628 (83.8)
0% < TN < 20%	57 (7.6)
TN ≥ 20%	64 (8.5)
Ki-67 index	
Ki-67 = 0%	639 (85.3)
0% < Ki-67 < 10%	66 (8.8)
Ki-67 ≥ 10%	44 (5.9)
Fuhrman grade	
I	239 (31.9)
II	324 (43.3)
III	140 (18.7)
IV	46 (6.1)
pT classification	
1a	229 (30.6)
1b	270 (36.0)
2a	130 (17.4)
2b	120 (16.0)
Tumor volume group	
V < 17 cm^3^	267 (35.6)
17cm^3^ ≤ V < 40 cm^3^	172 (23.0)
40cm^3^ ≤ V < 134 cm^3^	156 (20.8)
V ≥ 134 cm^3^	154 (20.6)
Median follow-up, mth	118.0 (85–155)
Local relapse	69 (9.2)
Metastatic progression	128 (17.1)
Death from cancer	196 (26.2)
Death	261 (34.8)

*Abbreviations*: *BMI* Body mass index

### Outline and calculation of TV

**Fig. 2:**
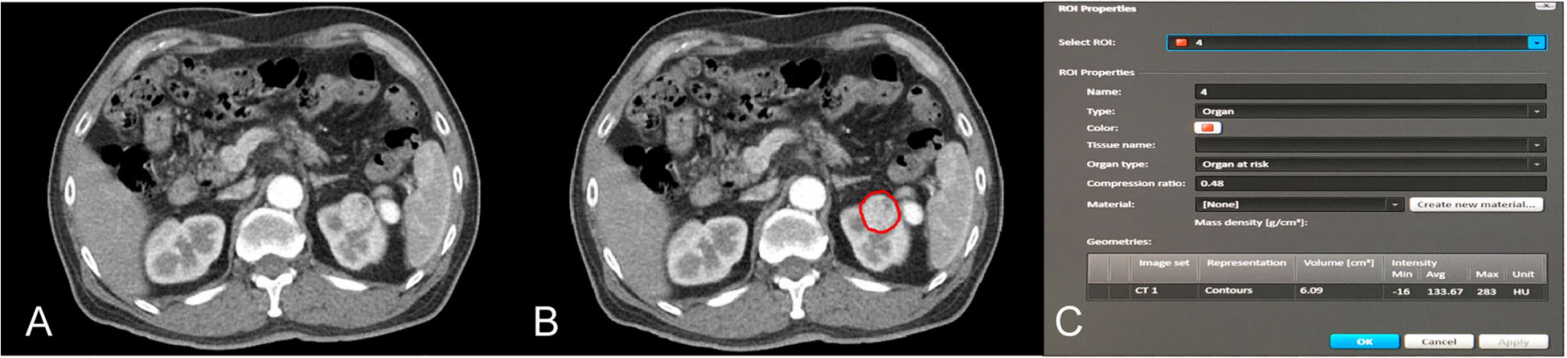
**a** Unmarked image; **b** Tumor contours were outlined; **c** After tumor contours were marked, tumor volume was automatically calculated by the 3D conformal radiotherapy planning system

The specific location, length and degree of invasion of ccRCC were determined by comprehensive analysis of preoperative CT images. Preoperative enhanced CT images were transmitted in digital format to the three-dimensional (3D) conformal radiotherapy planning system. Tumor contours of all cases were outlined by two experienced attendings in our center using the 3D conformal radiotherapy planning system, and TV was automatically calculated. Disagreements between the two attendings were settled by a third urologist (Fig. 2).

### Statistical analysis

Demographic features of patients and radiographic and pathological characteristics of renal masses were summarized. Baseline characteristics are presented using standard descriptive statistics: means ± standard deviations (SDs) for continuous variables with normal distribution; median (interquartile range) for continuous variables with non-normal distribution means; number (percentage) for categorical variables. Data analysis was conducted by using SPSS 19.0 (SPSS Inc., Chicago, IL, USA). Chi-square test was used to analyze qualitative variables while continuous variables were compared using Student’s t test. The mean values between groups were analyzed using analysis of variance, and the correlation between variables was tested by Spearman correlation analysis. The consistency test was used to evaluate the consistency on the 3D calculation of tumor volume between the two experienced attendings. The optimal cut-off level of TV for overall survival (OS) was determined using the web services-based Cutoff Finder (http://molpath.charite.de/cutoff/) [22]. There were a total of five methods for cutoff optimization in Cutoff Finder. The first method is based merely on the distribution of biomarker. Methods 2–4 deal with optimization of the correlation with a binary variable. Method 5 is used to optimize the correlation with survival variables. In this study, data containing information of TV, overall survival and survival status were uploaded and TV was assigned as biomarker. ROC curve was chosen as method and Waterfall plot was used to show the correct classification of the grouping. Univariable and multivariable regression models were used to explore the association between TV and oncological outcomes and clinicopathological data. Kaplan-Meier analysis was used to estimate the survival probabilities and log-rank test was used to determine the significance of OS, CSS, FFM and FFLR of localized ccRCC among various TV, Fuhrman grade and pT classification. Further, to estimate the accuracy of prognostic factors, we used receiver operating characteristic (ROC) curves to assess the prognostic effect of each risk factor for oncological outcome over 5 year with binomial estimation of confidence intervals (CI) of the area under the curve (AUC). We considered *P* < 0.05 as statistical significance.

## Results

### Patients and tumor characteristics

Sixty patients without complete CT/CT angiography data and 21 patients with incomplete follow-up were excluded. This left a final cohort of 749 ccRCC patients with CT/CT angiography data who underwent partial or radical nephrectomy at our center. There were 505 (67.4%) men. Mean age of all patients was 54.72 (18–72) years. Mean tumor diameter was 5.94 (0.60–14.10) cm. Partial nephrectomy (PN) was performed in 317 (42.3%) cases. The consistency test demonstrated that Kappa value was 0.910 and *P* value < 0.05, indicating that there was an excellent consistency on the 3D calculation of tumor volume between the two experienced attendings.

**Fig. 3:**
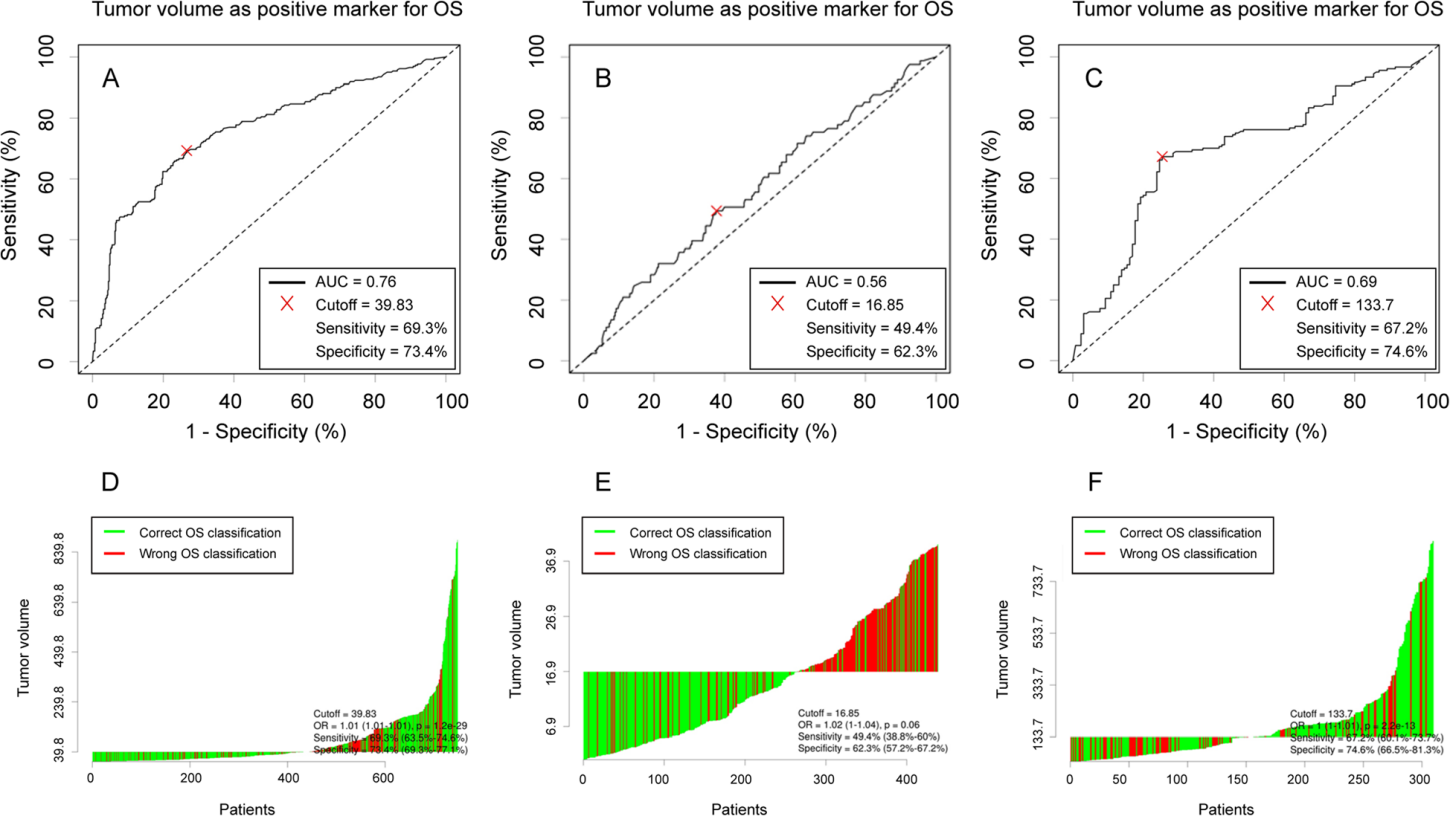
The ROC curves (**a**, **b**, **c**) and Waterfall plots (**d**, **e**, **f**) are presented. Three cutoffs were selected as 17, 40 and 134, which approximated to 16.85, 39.83 and 133.7, respectively. Tumor volume was graded as group 1 (V < 17 cm^3^), group 2 (17 ≤ V < 40 cm^3^), group 3 (40 ≤ V < 134 cm^3^) and group 4 (V ≥ 134 cm^3^)

The optimal cutoff level of TV for OS was determined by using ROC analysis, and the ROC curves and Waterfall plots are presented in Fig. 3. Firstly, the Cutoff Finder identified the optimal cut-off value as 40 cm^3^ and we classified patients into two groups according to the tumor volume of 40 cm^3^. Next, we further classified each of them into two groups and the Cutoff Finder identified the optimal cut-off value as 17 cm^3^ and 134 cm^3^, respectively. TV was graded as group 1 (TV < 17 cm^3^), group 2 (17 ≤ TV < 40 cm^3^), group 3 (40 ≤ TV < 134 cm^3^), and group 4 (TV ≥ 134 cm^3^) and there were 267 (35.6%), 172 (23.0%), 156 (20.8%), and 154 (20.6%) patients in each group, respectively. Patients and tumors characteristics are presented in Table 1.

### Oncological outcomes

**Fig. 4:**
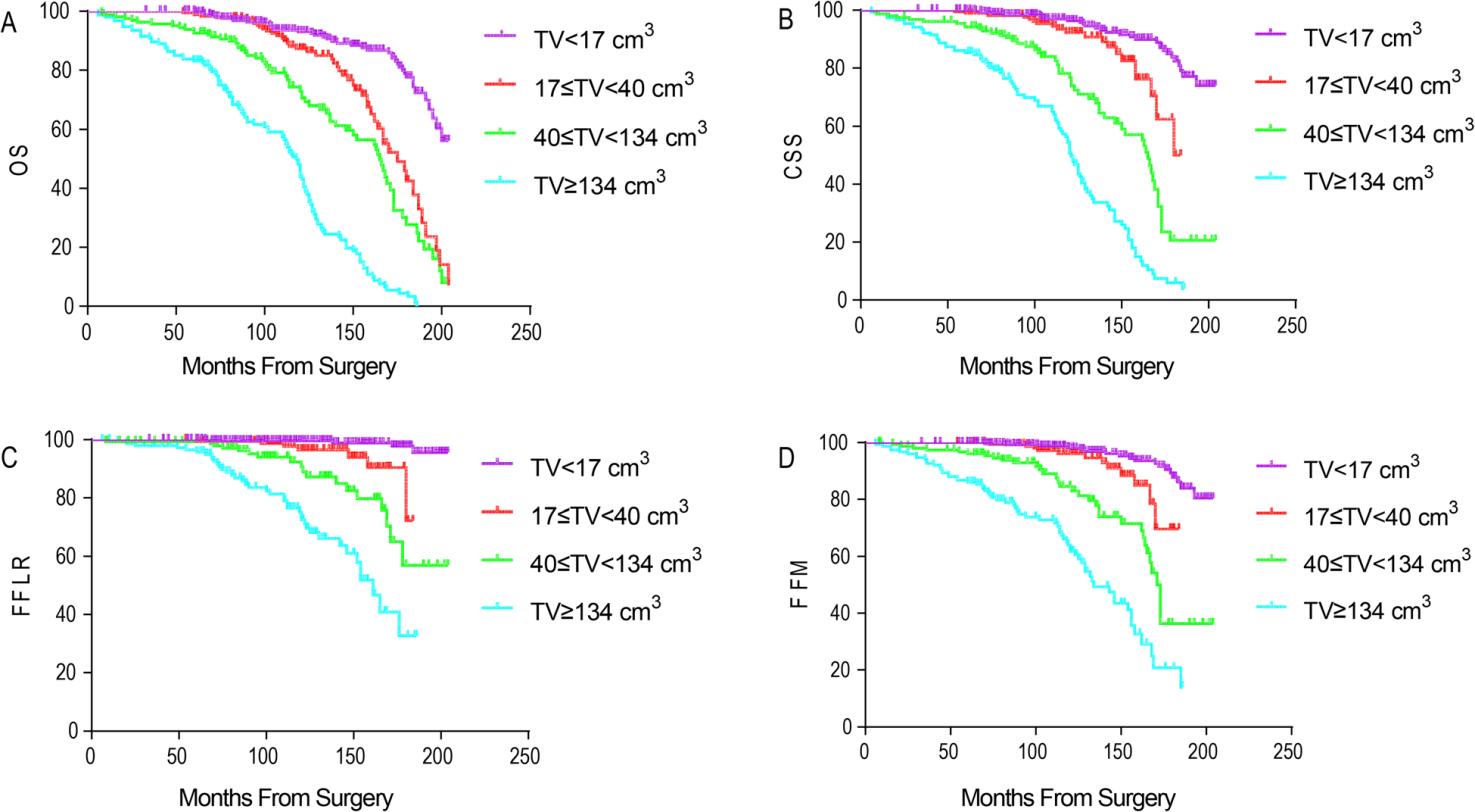
Kaplan–Meier curve analyses of OS (**a**), CSS (**b**), FFLR (**c**) and FFM (**d**) of ccRCC patients stratified by tumor volume (*P* < 0.05 for all)

**Fig. 5:**
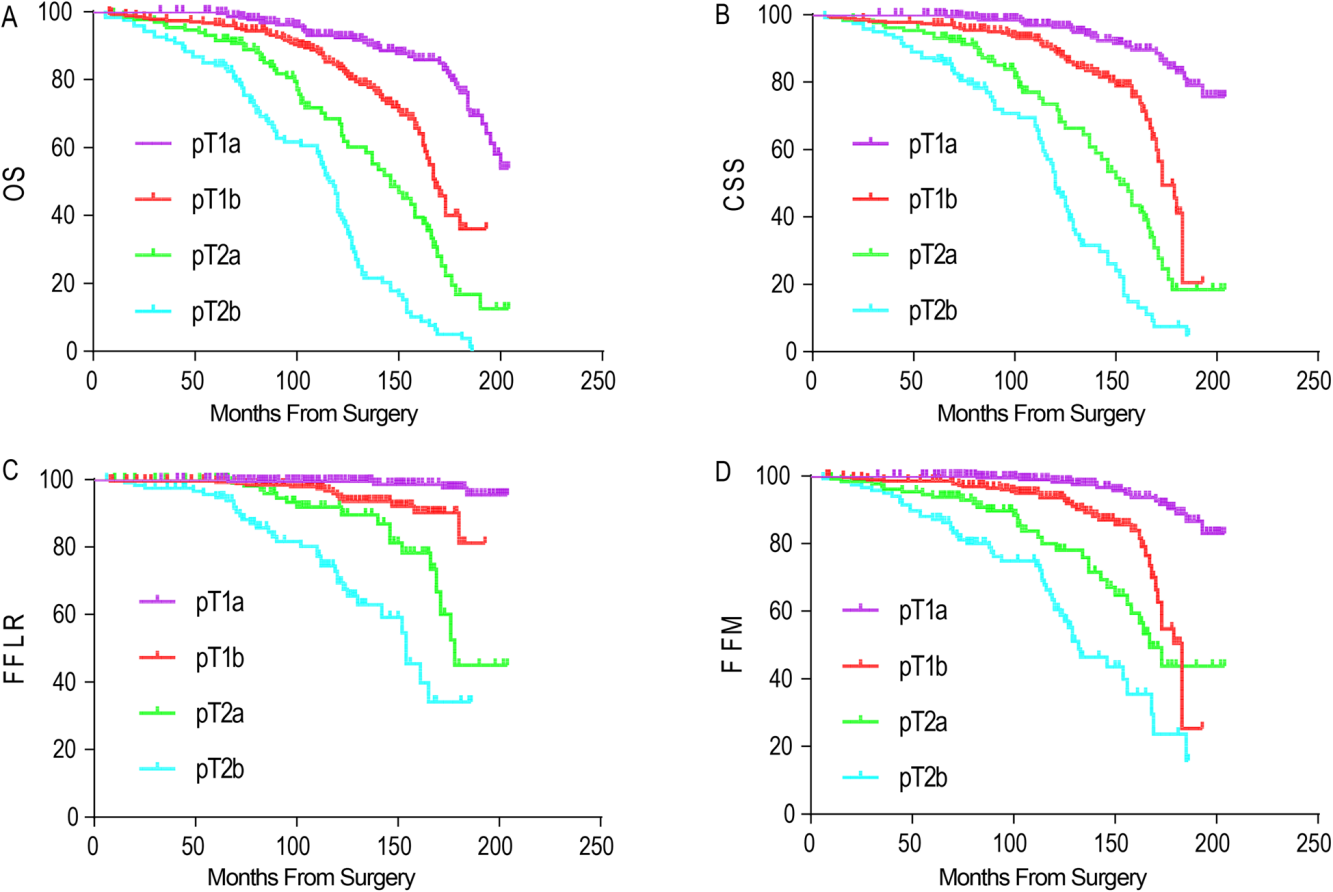
Kaplan–Meier curve analyses of OS (**a**), CSS (**b**), FFLR (**c**) and FFM (**d**) of ccRCC patients stratified by pT classification (*P* < 0.05 for all)

**Fig. 6:**
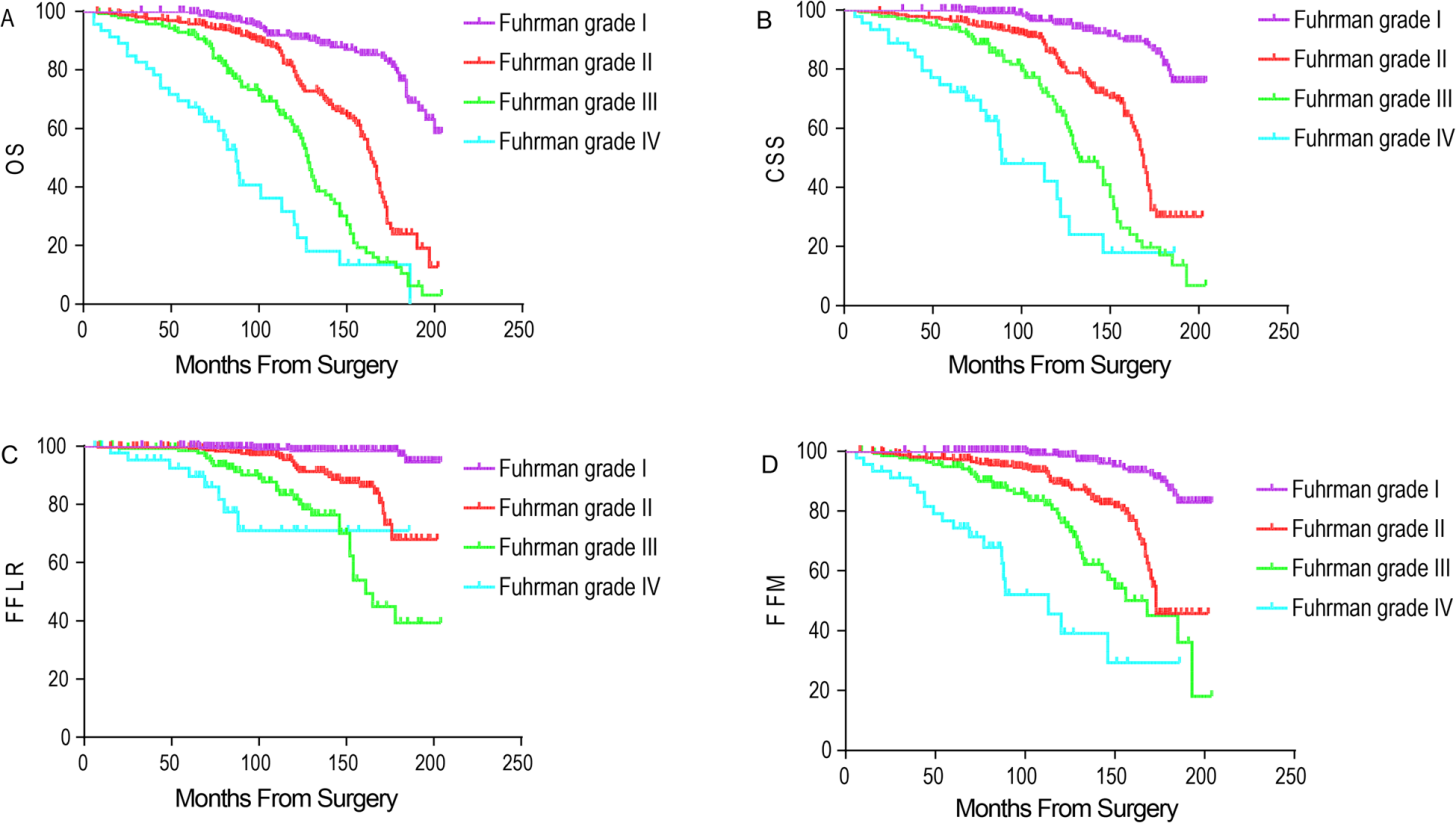
Kaplan–Meier curve analyses of OS (**a**), CSS (**b**), FFLR (**c**) and FFM (**d**) of ccRCC patients stratified by Fuhrman grade (*P* < 0.05 for all)

During follow-up, there were 65 deaths independent of RCC and 196 from RCC. There were 69 patients with local recurrence and 128 had metastatic progression. The OS of all patients at 5 and 10 years postoperatively were 94.6% [95% confidence interval (CI), 93.0–96.2%] and 77.1% (95% CI, 73.8–80.4%), respectively. Log rank test revealed that high TV, high Fuhrman grade and advanced pT classification were associated with poor OS, CSS, FFLR and FFM of patients after surgery (*P* < 0.001 for all) (Figs. 4, 5, 6).

Univariable Cox proportional hazards analysis showed that high Fuhrman grade, high TV and advanced pT classification were all associated with poor OS, CSS, FFLR and FFM of localized ccRCC after surgery, but tumor necrosis and Ki-67 did not predict the oncological outcome independently (*P* < 0.05 for all; Tables 2, 3, 4 and 5). In multivariable analysis, Fuhrman grade and TV has significant association with OS and CSS (*P* < 0.05 for all; Tables 2 and 3). Correlation between TV and FFLR was significant in the multivariable model (*P* < 0.05, Table 4), although univariable analysis showed that Fuhrman grade, pT classification and TV were all associated with FFLR of localized ccRCC after surgery. Multivariable analysis of the whole cohort of patients indicated that pT classification, TV and Fuhrman grade of ccRCC, has significant association with FFM (*P* < 0.05 for all, Table 5). The Cox model have been evaluated by the concordance index and have values of 0.78, suggesting relatively similar performance by utilizing R packages.

**Table 2:** Cox regression analysis of predicting factors for overall survival in 749 patients with localized clear cell renal cell carcinoma

Variable	Univariate analysis		Multivariate analysis	
	HR (95% CI)	*P* value	HR (95% CI)	*P* value
pT classification		< 0.001		0.123
(T1b vs. T1a)	2.79 (1.86–4.19)	< 0.001	1.08 (0.66–1.87)	0.401
(T2a vs. T1a)	5.56 (3.70–8.36)	< 0.001	1.24 (0.86–3.82)	0.525
(T2b vs. T1a)	12.92 (8.79–18.99)	< 0.001	2.45 (0.96–4.19)	0.442
Age(≥65 vs < 65)	0.93 (0.70–1.23)	0.927	0.82 (0.64–1.12)	0.658
Gender	0.89 (0.69–1.15)	0.362		
Fuhrman grade		< 0.001		0.001
(II vs. I)	3.62 (2.50–5.25)	< 0.001	1.21 (0.70–2.09)	0.494
(III vs. I)	8.17 (5.57–11.98)	< 0.001	1.60 (0.88–2.91)	0.123
(IV vs. I)	18.08 (11.12–29.40)	< 0.001	3.10 (1.57–6.12)	0.001
Smoking history	1.10 (0.86–1.40)	0.449		
Hypertension	1.01 (0.78–1.29)	0.970		
Diabetes	1.04 (0.74–1.47)	0.819		
Tumor necrosis extent		0.651		
0% < TN < 20% vs. TN = 0%	0.86 (0.51–1.45)	0.560		
TN ≥ 20% vs. TN = 0%	1.15 (0.77–1.73)	0.504		
Ki-67 index	–	0.507		
0% < Ki-67 < 10%vs. Ki-67 = 0%	1.13 (0.76–1.68)	0.537		
Ki-67 ≥ 10%vs. Ki-67 = 0%	1.29 (0.80–2.09)	0.296		
Tumor volume group		< 0.001		< 0.001
17cm^3^ ≤ V < 40 cm^3^ vs. V < 17 cm^3^	2.96 (1.88–4.65)	< 0.001	2.66 (1.54–4.60)	< 0.001
40cm^3^ ≤ V < 134 cm^3^ vs V < 17 cm^3^	5.08 (3.39–7.60)	< 0.001	4.25 (2.39–7.58)	< 0.001
V ≥ 134 cm^3^ vs V < 17 cm^3^	14.19 (9.80–20.56)	< 0.001	9.02 (5.06–16.08)	< 0.001
BMI(≥25 vs < 25)	0.93 (0.72–1.21)	0.589		
Sugical approach (open vs laparoscopic)	0.945 (0.74–1.21)	0.665		
Surgical modality (partial vs radical nephrectomy)	1.25 (0.97–1.60)	0.081		
Tumor location (left vs right kidney)	0.79 (0.62–1.00)	0.053		

*Abbreviations*: *BMI* Body mass index, *HR* Hazard ratio, *95% CI* 95% confidence interval

**Table 3:** Cox regression analysis of predicting factors for cancer-specifc survival in 749 patients with localized clear cell renal cell carcinoma

Variable	Univariate analysis		Multivariate analysis	
	HR (95% CI)	*P* value	HR (95% CI)	*P* value
pT classification		< 0.001		0.264
(T1b vs. T1a)	3.31 (2.00–5.47)	< 0.001	1.02 (0.86–1.52)	0.093
(T2a vs. T1a)	8.18 (4.99–13.42)	< 0.001	2.11 (0.89–4.07)	0.670
(T2b vs. T1a)	16.75 (10.40–26.97)	< 0.001	2.87 (0.97–4.73)	0.344
Age(≥65 vs < 65)	0.95 (0.69–1.32)	0.757	0.86 (0.61–1.25)	0.297
Gender	0.92 (0.68–1.24)	0.583		
Fuhrman grade		< 0.001		0.020
(II vs. I)	4.43 (2.82–6.95)	< 0.001	1.27 (0.63–2.55)	0.507
(III vs. I)	9.84 (6.18–15.66)	< 0.001	1.50 (0.71–3.17)	0.294
(IV vs. I)	21.51 (12.08–38.30)	< 0.001	2.84 (1.23–6.55)	0.015
Smoking history	1.28 (0.97–1.69)	0.087		
Hypertension	1.02 (0.77–1.37)	0.865		
Diabetes	1.02 (0.68–1.52)	0.922		
Tumor necrosis extent		0.867		
0% < TN < 20% vs. TN = 0%	1.02 (0.58–1.80)	0.941		
TN ≥ 20% vs. TN = 0%	1.14 (0.71–1.83)	0.593		
Ki-67 index		0.434		
0% < Ki-67 < 10%vs. Ki-67 = 0%	1.19 (0.76–1.87)	0.438		
Ki-67 ≥ 10%vs. Ki-67 = 0%	1.36 (0.79–2.35)	0.269		
Tumor volume group		< 0.001		< 0.001
17cm^3^ ≤ V < 40 cm^3^ vs. V < 17 cm^3^	2.19 (1.22–3.94)	0.009	1.93 (0.95–3.91)	0.069
40cm^3^ ≤ V < 134 cm^3^ vs V < 17 cm^3^	6.53 (4.09–10.42)	< 0.001	5.35 (2.64–10.87)	< 0.001
V ≥ 134 cm^3^ vs V < 17 cm^3^	16.36 (10.57–25.32)	< 0.001	10.95 (5.39–22.25)	< 0.001
BMI(≥25 vs < 25)	0.92 (0.68–1.24)	0.579		
Sugical approach (open vs laparoscopic)	0.96 (0.72–1.27)	0.753		
Surgical modality (partial vs radical nephrectomy)	0.92 (0.69–1.22)	0.555		
Tumor location (left vs right kidney)	0.79 (0.62–1.00)	0.053		

*Abbreviations*: *BMI* Body mass index, *HR* Hazard ratio, *95% CI* 95% confidence interval

**Table 4:** Cox regression analysis of predicting factors for freedom from local recurrence in 749 patients with localized clear cell renal cell carcinoma

Variable	Univariate analysis		Multivariate analysis	
	HR (95% CI)	*P* value	HR (95% CI)	*P* value
pT classification		< 0.001		0.629
(T1b vs. T1a)	5.46 (1.77–16.85)	0.003	1.10 (0.25–4.83)	0.965
(T2a vs. T1a)	15.94 (5.31–47.91)	< 0.001	2.56 (0.82–6.54)	0.271
(T2b vs. T1a)	43.16 (15.03–123.95)	< 0.001	2.24 (0.40–12.26)	0.213
Age(≥65 vs < 65)	0.75 (0.41–1.34)	0.326	0.74 (0.39–1.32)	0.258
Gender	0.81 (0.50–1.32)	0.396		
Fuhrman grade		< 0.001		0.300
(II vs. I)	7.10 (2.69–18.69)	0.001	0.89 (0.28–3.43)	0.137
(III vs. I)	21.80 (8.33–57.05)	0.001	1.98 (0.76–7.93)	0.488
(IV vs. I)	36.92 (11.84–115.11)	< 0.001	3.12 (0.96–11.32)	0.178
Smoking history	1.42 (0.89–2.28)	0.146		
Hypertension	0.95 (0.58–1.55)	0.827		
Diabetes	0.91 (0.45–1.83)	0.782		
Tumor necrosis extent		0.424		
0% < TN < 20% vs. TN = 0%	1.15 (0.46–2.87)	0.769		
TN ≥ 20% vs. TN = 0%	1.60 (0.79–3.23)	0.193		
Ki-67 index		0.210		
0% < Ki-67 < 10%vs. Ki-67 = 0%	1.79 (0.94–3.44)	0.078		
Ki-67 ≥ 10%vs. Ki-67 = 0%	1.17 (0.42–3.23)	0.764		
Tumor volume group		< 0.001		< 0.001
17cm^3^ ≤ V < 40 cm^3^ vs. V < 17 cm^3^	5.10 (1.47–17.71)	0.010	4.52 (1.31–15.42)	0.010
40cm^3^ ≤ V < 134 cm^3^ vs V < 17 cm^3^	15.75 (5.28–47.05)	< 0.001	13.25 (3.53–39.42)	< 0.001
V ≥ 134 cm^3^ vs V < 17 cm^3^	44.85 (15.74–127.84)	< 0.001	32.26 (12.32–117.65)	< 0.001
BMI(≥25 vs < 25)	0.76 (0.45–1.29)	0.313		
Sugical approach (open vs laparoscopic)	0.77 (0.47–1.26)	0.294		
Surgical modality (partial vs radical nephrectomy)	1.12 (0.70–1.79)	0.650		
Tumor location (left vs right kidney)	0.81 (0.51–1.31)	0.061		

*Abbreviations*: *BMI* Body mass index, *HR* Hazard ratio, *95% CI* 95% confidence interval

**Table 5:** Cox regression analysis of predicting factors for freedom from metastasis in 749 patients with localized clear cell renal cell carcinoma

Variable	Univariate analysis		Multivariate analysis	
	HR (95% CI)	*P* value	HR (95% CI)	*P* value
pT classification		< 0.001		0.048
(T1b vs. T1a)	4.10 (2.16–7.78)	0.001	2.29 (0.73–7.18)	0.155
(T2a vs. T1a)	8.65 (4.55–16.44)	< 0.001	1.04 (0.28–3.95)	0.949
(T2b vs. T1a)	19.22 (10.39–35.57)	< 0.001	0.71 (0.16–3.12)	0.651
Age(≥65 vs < 65)	1.06 (0.72–1.57)	0.769	1.02 (0.77–1.38)	0.614
Gender	0.90 (0.63–1.30)	0.587		
Fuhrman grade		< 0.001		0.004
(II vs. I)	4.70 (2.66–8.33)	< 0.001	1.15 (0.47–2.81)	0.757
(III vs. I)	9.70 (5.35–17.57)	< 0.001	1.52 (0.58–4.01)	0.398
(IV vs. I)	28.03 (14.11–55.69)	< 0.001	3.76 (1.32–10.74)	0.013
Smoking history	1.39 (0.98–1.96)	0.065		
Hypertension	1.10 (0.77–1.57)	0.609		
Diabetes	1.14 (0.71–1.84)	0.593		
tumor necrosis extent		0.986		
0% < TN < 20% vs. TN = 0%	1.06 (0.54–2.10)	0.865		
TN ≥ 20% vs. TN = 0%	1.00 (0.54–1.87)	0.993		
Ki-67 index		0.266		
0% < Ki-67 < 10%vs. Ki-67 = 0%	0.53 (0.25–1.14)	0.104		
Ki-67 ≥ 10%vs. Ki-67 = 0%	0.94 (0.44–2.02)	0.875		
Tumor volume group		< 0.001		<0.001
17cm^3^ ≤ V < 40 cm^3^ vs. V < 17 cm^3^	2.06 (0.98–4.35)	0.058	1.00 (0.33–3.08)	0.999
40cm^3^ ≤ V < 134 cm^3^ vs V < 17 cm^3^	6.45 (3.60–11.57)	0.001	4.44 (1.44–13.70)	0.009
V ≥ 134 cm^3^ vs V < 17 cm^3^	17.07 (9.91–29.42)	< 0.001	16.16 (4.27–61.10)	<0.001
BMI(≥25 vs < 25)	1.11 (0.78–1.60)	0.561		
Sugical approach (open vs laparoscopic)	0.99 (0.70–1.41)	0.970		
Surgical modality (partial vs radical nephrectomy)	1.05 (0.74–1.50)	0.772		
Tumor location (left vs right kidney)	0.73 (0.52–1.04)	0.080		

*Abbreviations*: *BMI* Body mass index, *HR* Hazard ratio, *95% CI* 95% confidence interval

**Fig. 7:**
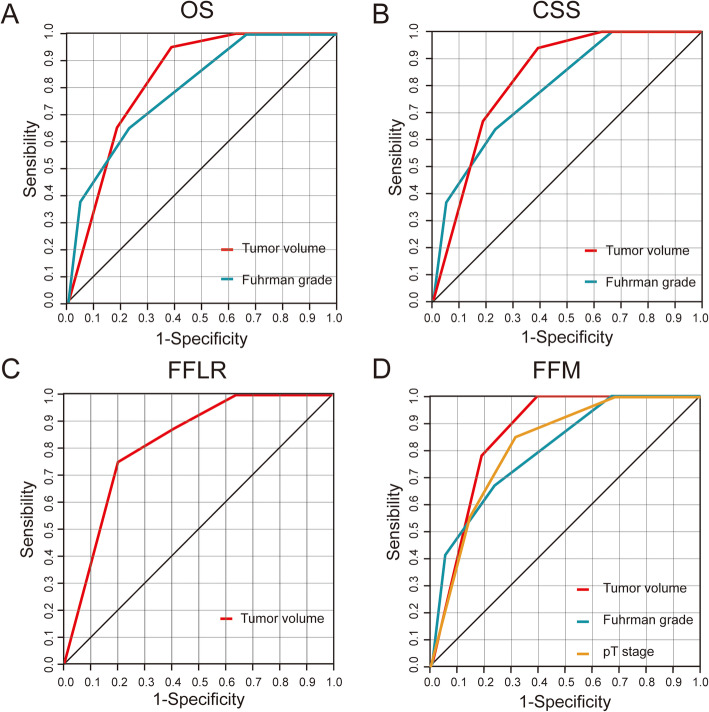
**a** The ROC curves of in diagnosis of OS; **b** ROC curves of in diagnosis of CSS; **c** ROC curves of in diagnosis of FFLR; **d** ROC curves of in diagnosis of FFM

The AUC of Fuhrman grade, pT classification and TV were compared for prognosis of localized ccRCC at 5 year postoperatively. For 5-year OS and CSS, the AUC of TV was higher than that of Fuhrman grade (0.832, 95%CI:0.787–0.876 vs. 0.799, 95%CI:0.735–0.864 and 0.829, 95%CI:0.780–0.879 vs 0.790, 95%CI:0.719–0.862, respectively) (Fig. 7a, b). The AUC of TV was 0.822 (95%CI:0.710–0.933) for prognosis of 5-year FFLR (Fig. 7c). The AUC of TV (0.864, 95%CI:0.823–0.906) for 5-year FFM was higher than that of Fuhrman grade (0.803, 95%CI:0.726–0.881) and pT classification (0.818, 95%CI:0.754–0.881) (Fig. 7d).

## Discussion

RCC patients have a favorable oncological outcome after surgical treatment; however, there are still 20–30% localized RCC patients have metastases after surgery [5, 23]. Shah et al. found in a multi-institutional study that 5.6% of localized ccRCC patients treated with PN had disease recurrence [24]. Other studies have shown that TNM stage, Fuhrman grade, necrosis, warm ischemia time, multifocality, and bilateral occurrence of carcinoma are the most useful prognostic factors [6, 11, 12, 25]. Currently, there are no ideal prognostic factors of oncological outcomes of localized ccRCC. We assessed TV of localized ccRCC and its association with clinicopathological features and oncological outcomes.

Previous studies have suggested that the measurement of TV is applied to evaluation of renal function, but only a few report on the relationship between oncological outcomes and TV of ccRCC [17–21]. Jorns et al. [26]. concluded that TV could provide valuable prognostic information for patients with pT1a ccRCC rather than pT1b ccRCC. The researchers believed that the accuracy of TV determination using radiological imaging is of great importance for patient management. Different applications have been used to view and analyze CT cross-sectional images, such as syngo Studio imaging software [17] and OncoCare CT oncology application beta software [18]. But these equations are relatively complex and not convenient for clinical application. Jorns et al. [26]. calculated TV using volume equation of ellipsoid [i.e.π/6 (length×width×height)]. However, most solid tumors are not regular spheres or ellipsoids. In this study, we introduced a more common method to calculate TV, namely, the 3D conformal radiotherapy planning system, which is widely used in the field of radiotherapy [27]. Volumetric measurements were made on enhanced CT images, in which RCCs were visualized with high resolution. This planning system is closer to the actual shape of the lesion, rather than simply overlaying the length × width × height.

Pathological tumor classification (pT), Fuhrman grade and other factors have been considered as the most useful oncological outcome prognostic factors in patients with RCC [28]. TNM staging is the most important index to reflect tumor progression. It is the basis for judging the prognosis of ccRCC and making the correct treatment decisions [29]. However, Lee et al. [30] showed that pT classification after PN is not a significant prognostic factor of CSS, OS or RFS in patients with small renal mass. In a recent study, most pT1a tumors recurred after 5 years [31]. Remarkably, univariable Cox proportional hazards analysis showed that pT classification has significant association with CSS, OS and FFLR. However, multivariable analysis further demonstrated that pT classification was not an independent prognostic factor for CSS, OS and FFLR. We think that this result was due to the relatively high OS of patients in our study, this conclusion needs to be further verified by larger sample and multi-center study.

Fuhrman grading system is one of the most extensively used histological prognostic factors and correlates with survival [29]. Tsui et al. reported 5-year survival rates of patients with Fuhrman grade 1, 2, and 3/4 was 89, 65 and 46.1%, respectively [32]. However, another study concluded that univariable analyses did not reveal that Fuhrman grade is a significant mortality risk factor for nonmetastatic RCC patients [13]. In our study, survival analysis demonstrated that higher Fuhrman grade has negative association with OS, CSS, FFLR and FFM for localized ccRCC after surgery. Multivariable Cox model showed that Fuhrman grade is a prognostic factor for OS and CSS but for FFLR and FFM.

A previous study conducted by Jorns and his colleagues demonstrated that when compared with tumor diameter, RCC TV of three dimension was of greater value in predicting prognosis for pT1a ccRCC patients, suggesting that RCC TV is a better indicator of actual tumor burden better than tumor diameter [26]. Meanwhile, Thiel et al. [33] evaluated 2180 patients’ data and reported that the traditional way of using the largest RCC tumor diameter for staging was of poor value in predicting real TV. They proposed that new prognostic algorithms or proper staging systems should examine the use of real solid TV rather than largest tumor diameter. With the application of artificial intelligence in medical imaging, computers are becoming more and more sophisticated in identifying tumor profiles and calculating various parameters [34, 35]. The measurement of TV is becoming easier and more accurate, which will facilitate the routine clinical measurement of TV [36].

As is known to us, this is the first report showing an association between TV and oncological outcomes of localized ccRCC. Here are some limitations of our study. Firstly, the short follow-up, retrospective, single-center characteristic and limited number of patients were the major limitations. Considering that we merely used Cutoff Finder and ROC method to identify optimal cutoff value without external validation, performance will be biased. Hence, the conclusions and the cutoff values require further external validation by prospective clinical studies with larger sample size and multicenter. Moreover, the number of patients with local tumor recurrence and metastasis were small. This may have affected the real relationship between Fuhrman grade, pT classification, TV and oncological outcome. Finally, we only selected patients with unifocal, unilateral pT1–2 ccRCCs, excluding metastatic and lymph node-positive patients at the time of diagnosis. Therefore, no conclusion can be drawn on pT3–4, lymph node-positive and metastatic ccRCC patients.

## Conclusions

In conclusion, higher TVs were all independent prognostic factors for poorer OS, CSS, FFLR and FFM of localized ccRCC following surgery. In comparison with other prognostic factors including Fuhrman grade or pT classification, TV is of more value for prognostic prediction of oncological outcome of localized ccRCC. TV could be a new prognostic factor of oncological outcome of localized ccRCC, which might contribute to tailored follow-up or management strategies.

## Data Availability

The datasets used and analyzed during the current study available from the corresponding author on reasonable request.

## Abbreviations

3D: Three-dimensional
AUC: The area under the ROC curves
ccRCC: Clear cell renal cell carcinoma
CI: Confidence interval
CSS: Cancer-specific survival
CT: Computed tomography
OS: Overall survival
FFM: Freedom from metastasis
FFLR: Freedom from local recurrence
pT classification: Pathological tumor classification
RCC: Renal cell carcinoma
ROC: Receiver operating characteristic curves
SDs: Standard deviations
TV: Tumor volume
